# The Huxley Lecture and Charing Cross Hospital Dinner

**Published:** 1912-11-09

**Authors:** 


### The Huxley Lecture and Charing Cross Hospital Dinner.

The Huxley Lecture was delivered on October 31 in the large out-patient hall of Charing Cross Hospital by Professor Simon Flexner, the director of the Rockefeller Institute of New York. A very large audience assembled to honour the distinguished lecturer, who was introduced by Sir William Osler. The address was entitled "Some Problems in Infection and its Control," and dealt principally with the subject of infantile paralysis or poliomyelitis.

In the evening the annual dinner of the past and present students of Charing Cross Hospital took place in the Adelaide Hall opposite the hospital. Professor Flexner, as the principal guest, again played a prominent part and made his third speech that day in singularly happy vein as one of the responders to the toast proposed by Dr. James Galloway. The chair was taken by Dr. F. W. Mott, who had many happy things to say of the school and the hospital, and strongly advocated the proposed institution of a diploma in Psychiatry, following the example of the D.P.H., the D.T.M., and the diploma in Ophthalmology. Principal Headlam, whose resignation had that day been announced, referred to the action of the Board of Education in respect of King's College and congratulated Charing Cross Hospital on its progressive policy. The dinner was largely attended.

